# A study of the reporting of patient and public involvement and engagement (PPIE) in orthodontic research

**DOI:** 10.1177/1465312520968574

**Published:** 2020-11-05

**Authors:** Veena A Patel, Jonathan Shelswell, Neil Hillyard, Sue Pavitt, Sophy K Barber

**Affiliations:** 1 Leeds Dental Institute, Leeds Teaching Hospital NHS Trust, Leeds, UK; 2 Hillyard MacDonald, Inverness, UK; 3 Dental Translational & Clinical Research Unit, University of Leeds, Leeds, West Yorkshire, UK; 4 Orthodontic Department, University of Leeds, Leeds, UK

**Keywords:** patient, public, involvement, engagement, methodology, research, orthodontics, study

## Abstract

**Introduction:**

Patient and public involvement and engagement (PPIE) in research is an essential component of high-quality research. Patients and the public can identify which research topics are most relevant to them, contribute to study design, and interpretation and dissemination of findings. While inclusion of PPIE is widely adopted in medical research, awareness within the dental research community is more limited.

**Aim:**

To examine patient and public involvement and engagement in orthodontic research activity.

**Design:**

Identification and appraisal of use of PPIE in orthodontic research reporting and funding applications using a systematic approach.

**Methods:**

Three sources of information were examined: (1) research articles published between September 2018 and September 2019 in four major orthodontic journals. Articles were examined for reported PPIE; (2) common funding bodies for orthodontic research were assessed to establish whether PPIE was mandated (National Institute for Health Research, Medical Research Council, Wellcome Trust, Chief Scientist Office (Scotland), Health and Care Research Wales, British Orthodontic Society Foundation, Royal College of Surgeons and CLEFT); and (3) publication guidance for authors in these journals was examined to identify whether reporting of PPIE was included.

**Results:**

Of the 363 research articles, 2 (0.6%) mention patient/public involvement. None of the 363 research articles mention patient/public engagement. Of nine funding bodies, 2 (22%) request evidence of patient/public involvement as a condition of receiving funding with one (11%) expecting evidence of public engagement to be provided as a condition of receiving funding. None of the four major orthodontic journals include patient/public involvement and/or engagement in their guidance for authors.

**Conclusion:**

There is currently: (1) a notable lack of reporting of PPIE in orthodontic research; (2) variability in the requirements of funding bodies for researchers to include PPIE in funding applications and throughout the research process; and (3) no stipulation in journals’ instructions for authors.

## Introduction

Patient and public involvement (PPI) in research refers to ‘*research being carried out “with” or “by” the public rather than “to” or “about” or “for” them*’ (National Institute for Health Research [NIHR] Central Commissioning Facility [CCF], 2019). Patient and public engagement (PaPE) describes activities that encourage the findings and benefits of research to be shared with the patients/the public (National Co-ordinating Centre for Public Engagement [NCCPE], 2019). Patient and public involvement and engagement (PPIE) in research is an essential component of high-quality research; patients and the public can contribute in a number of ways (Table 1) to help identify which research topics are most relevant, to improve study design and delivery, and to identify effective methods for disseminating research to the public (Staley, 2009, 2015). PPIE aims to create a mutually beneficial relationship between researchers, patients and the public.

**Table 1.** Examples of patient and public involvement and engagement activities in research (NCCPE, 2019; NIHR CCF, 2019; Staley, 2015, 2019).

Patient and public involvement in research design	• Establishing research priorities • Identifying and tailoring research questions to maximise patient benefit • Co-investigator of a research study, supporting grant development (recognised as co-applicant on grant) • Support operations and logistic planning so not too onerous for participants • Assisting communication with patients and public, e.g. writing lay summaries
Patient and public involvement in research delivery	• Membership of study management and steering groups • Guidance on recruitment strategies and communication with (potential) participants • Reviewing patient-facing documents, e.g. participant information sheets, consent forms • Review analysis and interpretation of findings
Patient and public involvement in research dissemination	• Content development for dissemination • Contribute to dissemination strategy
Patient and public engagement	• Public engagement events and resources that aid dissemination of research findings • Development of end of study lay summaries • Sharing research findings on social media and with patient/public forums, support groups, relevant charities and networks.

PPIE in the UK is increasingly important to justify funding and maximise the value and impact of research. The two-way conversation that public engagement facilitates is one which builds trust between researchers and the public and helps to build understanding and appreciation of the research which is conducted. It also helps researchers to shape their projects to meet the needs and expectations of the public (NCCPE, 2019; NIHR CCF, 2019). The evidence base for PPIE has grown in recent years and its inclusion is becoming increasingly prevalent in medical research (Boivin et al., 2018; Regan de Bere et al., 2016). However, awareness and uptake in the dental research community appears slower (Needleman, 2014). This study idea arose from discussions about PPIE with Mr Neil Hillyard, an orthodontic patient who was a founding member of British Orthodontic Society patient panel and is the author of the ukadultbraces.co.uk blog.

## Aim

The aim of the present study was to examine patient and public involvement and engagement in orthodontic research.

## Design

Orthodontic literature and funders of orthodontic research were appraised for use of PPIE using a systematic approach. A patient contributor (NH) advised on the purpose and analysis of this study.

## Methods

Three sources of information were examined:

1. Research articles published between September 2018 and September 2019 in four orthodontic journals (*Journal of Orthodontics, American Journal of Orthodontics and Dentofacial Orthopaedics, The Angle Orthodontist* and *European Journal of Orthodontics*). These were identified by assessing each article in each journal electronically to see whether it fulfilled the eligibility criteria. The eligibility criteria included any original research articles and systematic reviews that had a structure of background, methods, results, conclusion and discussion or similar. There was exclusion of case reports, case series, expert opinion papers, audits, service evaluation, letters to the editor and other obvious non-research articles. Each article was independently judged against the criteria by two authors (JS and VAP) and any disagreements were resolved by a third author (SB). All research articles fulfilling the eligibility criteria were included for analysis. Corresponding authors of the papers included in the analysis were emailed to investigate whether they used PPIE but did not report it in the final publication. A single email was sent and authors asked to respond within two weeks.
2. The websites of common funding bodies for orthodontic research were assessed to establish whether PPIE was mandated—NIHR, Medical Research Council (MRC), Wellcome Trust, Chief Scientist Office (Scotland) (CSO), Health and Care Research Wales (HCRW), British Orthodontic Society Foundation (BOSF), Royal College of Surgeons (RCS) and CLEFT.
3. Publication guidance for authors in these major orthodontic journals was examined to identify whether reporting of PPIE was included.

Judgement of sources:

- Sources were judged against pre-defined criteria (Table 2) by two independent reviewers (VAP and JS). Any disagreements were discussed with a third reviewer (SB). Where the criteria were scored as ‘Yes’, details were recorded to allow further analysis.
- Where PPI was reported in a paper, the quality of PPI reporting was evaluated using the Guidance for Reporting Involvement of Patients and Public (GRIPP2) checklist (Staniszewska et al., 2017).

**Table 2.** Criteria used to judge requirements for, and reporting of, patient and public involvement and engagement (PPIE; this was devised by the authors for the purpose of the study).

Source	Judgement criteria	Scoring
Research article	Planned use of PPIE in:	
	• Research design	Yes / No
	• Research delivery	Yes / No
	• Research dissemination	Yes / No
	Actual use of PPIE in:	
	• Research design	Yes / No
	• Research delivery	Yes / No
	• Research dissemination	Yes / No
	Discussion of impact of PPIE	Yes / No
Author guidelines	Patient and public involvement	
	• Included in guidelines for authors	Yes / No
	• Mandated for publication	Yes / No
	Patient and public engagement	
	• Included in guidelines for authors	Yes / No
	• Mandated for publication	Yes / No
	Does the journal request lay summaries of research?	Yes / No
Funding bodies	Patient and public involvement	
	• Included on website	Yes / No
	• Mandated for funding	Yes / No
	Patient and public engagement	
	• Included on website	Yes / No
	• Mandated for funding	Yes / No
	Do lay representatives contribute to funding decisions?	Yes / No
	Does the funder publish lay summaries of funded studies?	Yes / No

## Results

### Reporting of PPIE in published research

**Figure 1.**
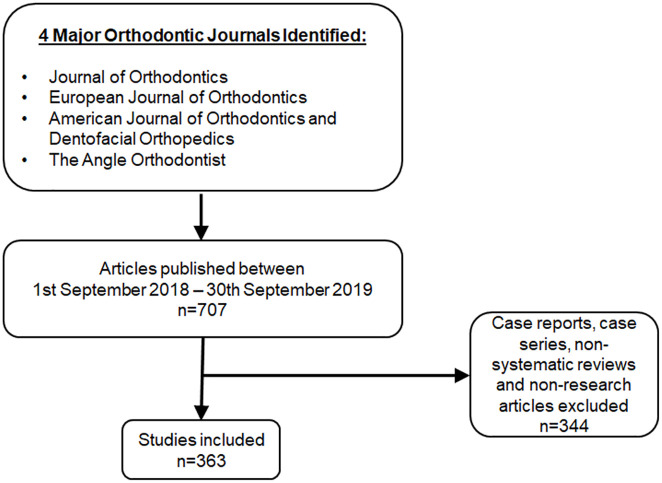
Summary of the methods for identifying studies to include in the analysis.

From the four journals examined, 707 articles were identified, of which 363 were eligible for inclusion (Figure 1). Of the 363 research articles, only 2 (0.6%) report PPI and both were in the design of patient-facing materials (Ahn et al., 2019; Chambers and Zitterkopf, 2019). None of the 363 research articles reported PPI in the other aspects of research design and delivery. Neither of the papers that did utilise PPI, reported their use of PPI against the GRIPP2 checklist. Since PPI was not the main focus of either of the two studies, we have used the GRIPP2 short form to analyse the two papers against the criteria (Table 3).

**Table 3.** GRIPP2 short form applied to both papers which mention patient and public involvement (PPI).

GRIPP2 criteria	Paper 1 (Ahn et al., 2019)	Paper 2 (Chambers and Zitterkopf, 2019)
Aims of PPI	Not stated.	Not stated.
PPI methods	‘The mind map was also shown to a group of patients to obtain lay opinion’	‘The first version of a survey was tested in a focus group of six patients’
Study results (outcomes of PPI)	Positive feedback, including that it was easy to read and understand, was obtained	The survey was ‘revised accordingly’ after the focus group
Discussions and conclusions	None given	None given
Reflections/critical perspective	None given	None given

Authors for 52 (14%) of the 363 studies replied to our email request regarding the use of PPIE and, of these, only 2 (4%) reported use of PPI. One of these authors has previously been identified, as the use of PPI was reported in their paper (Chambers and Zitterkopf, 2019). The second reply was from a co-author of the present study (SB), who had used PPI in another study published in the *Journal of Orthodontics* but had not reported it (Barber et al., 2019). The author provided a summary of their use and experience of PPI (Box 1).

**Box 1.** Co-author SB’s experience of using patient and public involvement (PPI) in research.

The article in the *Journal of Orthodontics* (*JO*) relates to part of the work undertaken within my PhD, which was an NIHR-funded Doctoral Research Fellowship. The NIHR mandate PPI as part of the funding application process and, as such, patients and parents contributed as PPI collaborators at various points throughout the four-year study. This was my first experience of using PPI in research and, although challenging at times, I found the experience enjoyable and I really felt the research benefitted from it. PPI contribution in my research included: forming the research question; design, planning and conduct of the study; and data analysis. I found involving PPI collaborators in these stages provided a different perspective on the research, for example, understanding how young people might understand hypodontia and the best approach for asking questions to get the information I needed. Engaging with parent helped me pre-empt potential issues for recruitment and data collection, and it also provided an alternative perspective on the meaning of my findings during analysis. The main challenges I faced in involving young people and parents as collaborators were recruitment and training, as many of the young people were at a transitional stage of their own lives within the research period (for example, moving from school to college or university) and then practical issues and logistics when trying to arrange meetings to fit around school and work. As this work was part of my PhD, I was fortunate to have a supervisor with expertise in PPI and she supported me with identifying and training PPI collaborators. I also used many of the INVOLVE resources. To make organisation easier I often met PPI collaborators individually, rather than as a group, which limited the scope for group interaction but increased flexibility for arranging meetings. I did not describe the use of PPI in my *JO* article due to limitations on word count and the challenge of explaining it in a useful way for readers. As a novice in PPI, I found it can be difficult to quantify how PPI shaped and improved the research. However, having completed my PhD and reflected on the use of PPI, I strongly feel that reporting PPI is important to promote its stakeholder engagement in the research process, and in future publications, I will strive to report this as a key aspect of methodological quality.

None of the 363 research articles reported PaPE in disseminating the research findings to the public. Corresponding authors of 5 (9.6%) of the 52 studies for which we received replies confirmed their use of PaPE. All five authors exclusively reported posting their research on social media platforms but of these, only 2 (40%) stated that they provided a plain English summary to accompany the post.

### PPIE requirements of funders

PPIE requirements of the funders is summarised in Table 4. Two (22%) of the nine funders (NIHR, 2019; HCRW, 2019) request evidence of PPI as a condition of receiving funding, while one funder (11%) (CSO, 2019) expects evidence of PaPE as a condition of receiving funding. Three funders (33%) (CSO, 2019; MRC, 2019; NIHR, 2019) clearly state a requirement for lay or plain English summaries to be provided in the publication of research.

**Table 4.** Requirement for PPIE as reported on the websites of the nine funding bodies.

Funding body	Type of research funded	Patient and public involvement mandated?	Patient and public engagement mandated?
Association of Medical Research Charities (AMRC, 2019)	A group of over 140 charities that fund medical and healthcare research	No – but they have a briefing document about the patient voice in medical and healthcare research	No – but they have a briefing document on the patient voice in medical and healthcare research
British Orthodontic Society Foundation (BOSF, 2019)	Evidence-based orthodontic care and health benefits of treatment, fund projects that improve orthodontic teaching	No	No
Chief Scientist Office Scotland (CSO, 2019)	Health Services Research, Health Economics, Hearing Research, Nursing and Allied Health Professions, Social and Public Health Sciences, Scottish Collaboration for Public Health Research Policy	No – but it is encouraged. ‘Over the last 18 months, CSO have been part of a partnership developing a set of standards and indicators for public involvement in research.’	Yes – ‘The grant holder and/or Chief Investigator and/or co-Investigators are expected to participate in activities which seek to raise awareness of science amongst lay audiences. Research active NHS organisations are expected to develop and deliver their own communication strategies and in some cases, if relevant, local Investigators might be able to involve themselves with those communication initiatives.’
CLEFT (CLEFT, 2019)	Genetics, speech therapy, orthodontics, surgery	No guidance readily available	No guidance readily available
Health and Care Research Wales (HCRW, 2019)	Health and social care research	Yes – ‘Researchers applying to HCRW funding schemes are required to demonstrate public involvement in their applications.’	No.
Medical Research Council (MRC, 2019)	Prevention and early detection, precision medicine, multi-morbidities, advanced therapies, mental health, antimicrobial resistance, global health	No	No – but it is encouraged; ‘MRC-funded scientists are encouraged to participate in engagement activities. Sharing our research with the public who fund it and the wider world is a crucial part of the MRC mission.’
National Institute for Health Research (NIHR, 2019)	Innovation and product development, public health, efficacy and mechanism research, evaluative research including large-scale, pragmatic clinical trials	Yes. Included on the checklist for an NIHR Stage 1 application; ‘Appropriate and relevant involvement of patients and the public.’	No - but it is encouraged; ‘We encourage all applicants for NIHR funding to prospectively (and realistically) consider how to engage with and involve research users at every stage of the research process, considering how users might benefit from the research.’
Royal College of Surgeons (RCS, 2019)	Surgical research and the development of new operative techniques	No – but they have a Patient and Lay group who give patients and the public a voice into the day-to-day running of the RCS	No
Wellcome Trust (Wellcome, 2019)	Biomedical science, population health, product development and applied research, humanities and social science, public engagement and creative industries	No	No- but it is encouraged and grants are available for use in engaging the public. ‘To help, we offer support for scientists and academics who want to engage the public with their research.’

### PPIE requirements of journals

None of the four orthodontic journals or their publishers include PPIE in their guidance for authors (American Journal of Orthodontics and Dentofacial Orthopedics, n.d.; The Angle Orthodontist, n.d.; Elsevier, n.d.; European Journal of Orthodontics, n.d.; Journal of Orthodontics, n.d.; Oxford University Press, n.d.; SAGE Journals, n.d.).

## Discussion

The results of this study suggest a lack of reporting of PPIE in orthodontic research, an absence of prompting in guidance from journals and publishers, and inconsistent requirements from funding bodies regarding the need for, and reporting of, PPIE. Our results substantiate findings from a systematic review of the quality of reporting PPI in surgical specialties, which found that only a limited number of studies reported use of PPI (Jones et al., 2015). We were not able to find any studies examining the reported use of public engagement in health research.

There are many potential detrimental consequences of not employing PPIE in research. Chalmers and Glasziou (2009) estimated that 85% of all clinical research is wasted, even when huge investments and public funding have been utilised. The authors also deduced that there are four research practices which could result in such waste: (1) prioritising research questions which are not relevant to the public (as well as clinicians); (2) conducting unnecessary or inappropriate studies or study designs; (3) failing to publish research findings; and (4) selective reporting of research findings. NH provided a patient’s perspective on these findings (Box 2), which highlights both the need and drivers for PPIE, but also potential challenges.

**Box 2.** Patient perspective (NH) of the review findings.

‘*I felt that this was very interesting and certainly highlighted the enormous amount of work that needs to take place for PPIE to be commonplace. No doubt, linking the requirement to funding will be a strong motivator as will being part of the conditions of publication. From a personal (patient) perspective, I feel it is essential that patients have a voice during the research both with the design and, in a sense, checking the results and conclusions. I would imagine recruitment would be a challenge, but this is often down to the relationship with the patient and the expectations of requirements of the role. I think part of the problem is lack of clarity of what is involved and the timescale for their contribution. In my experience, research takes a while and expectations need to be set that involvement can be for a length of time.*’

Alongside impacting on research quality, a lack of PPIE reporting means that others cannot learn from the other researcher’s experience and it is difficult to evaluate the effectiveness of different approaches to PPIE. Improved reporting of PPIE will lead to a more expansive and high-quality evidence base for PPIE, allowing us to better understand its uses and effectiveness (Staniszewska et al., 2017). The EQUATOR network (EQUATOR, 2019), which is an international initiative to promote transparent and accurate reporting of health research, includes the GRIPP2. However, no guidelines on the reporting of PaPE could be identified and the International Committee of Medical Journal Editors (ICMJE, 2019) recommendations do not provide guidance on the reporting of PPI or PaPE. GRIPP2 reporting guidelines were published in 2017 and these are the first evidence-based, international, consensus community-informed guideline of its kind in health and social care research. The guidance includes two checklists: one is a short version that can be used to report PPI in any study; and the other is a long form which should be used where PPI is the focus of the study and can be used to structure the entire paper. The forms can be used prospectively to guide the use of PPI and retrospectively to help structure the reporting of PPI (Staniszewska et al., 2017). Perhaps a requirement to report use of any PPIE could also be included in existing checklists for reporting research papers such as CONSORT, PRISMA and ROBINS-I.

It may be the case that many researchers are using PPI or PaPE in their research but are not reporting it. Indeed, one of the research papers reviewed in this study was undertaken by one of the authors of the present article and although the examined study (Barber et al., 2019) was funded by the NIHR and used extensive PPI, this is not reported in the paper. Of the 363 studies analysed, 161 were funded and this included funding from the NIHR, suggesting there will be other studies that used PPI but did not report it explicitly. However, in this study, although only 14% of authors replied to an email regarding use of PPIE, the low rates of PPIE utilisation identified within this sample correspond with the lack of reporting. The *British Medical Journal* described PPI reporting before and after a journal policy was introduced. The policy required authors to write if and how PPI was used, and this concluded that sporadic reporting of PPI was not only due to a lack of reporting but also due a lack of actual PPI activity and/or unwillingness to report unsuccessful PPI (Price et al., 2018).

Recommendations for increasing the use and reporting of PPIE are given in Box 3. A number of concerns may be raised in response to these recommendations: certain types of studies, such as laboratory studies, are too conceptual for laypeople to understand; if publishers and funding bodies make PPI or PaPE a compulsory requirement, this may limit the research that can be undertaken by those who may not have funding and support available to them; PPI may become tokenistic and lose any value (Domecq et al., 2014). The counter to this is that research with an immediate or eventual clinical impact should be able to be translated into a format that is accessible to a lay audience, and if patients and the public do not believe that a project is worth pursuing, then perhaps the proposed benefits are not adequately explained, or more importantly, are not valued. Capturing patient or public perspective may mean less money is wasted and resources are channelled into research that is deemed to be more useful (Boote et al., 2014).

**Box 3.** Recommendations for improving the use and reporting of patient and public involvement and engagement (PPIE) in orthodontic research.

Development of PPI networks • Formation of patient/public stakeholder groups for general and specialist dental research, for example, through universities or orthodontic societies. This would overcome some of the issues around recruitment, training, support and access to PPI. • Support, training and mentorship for researchers who are new to PPIE from people with experience of PPIE. Funders • More consistent and explicit requirement for PPIE across funders. • Small grants to support PPI during research design in preparation for funding application. • Support for evaluating PPIE to develop an evidence-base for which methods work best for different types of studies, populations and research settings. Journals • Inclusion of PPIE explicitly in journal’s guidance for authors to promote greater reporting. • Request for a lay summary alongside the main scientific manuscript for published research to improve accessibility for patients and the public. National bodies • Encouraging orthodontic patients to become PPI contributors via national bodies that promote volunteering, e.g. National Citizen Service, Duke of Edinburgh, National Council for Voluntary organisations, or as a valid volunteering option for potential medical and dental students.

INVOLVE is a national advisory board funded by the NIHR, which aims to support the use of public involvement in the NHS and health and social care research. INVOLVE provide freely accessible resources to support researchers and this is recommended as a starting point for researchers interested in PPIE. Furthermore, journals dedicated to PPIE, such as BioMed Central’s *Research Involvement and Engagement* and the National Coordinating Centre for Public Engagement’s *Research for All* may be a useful source for inspiration and shared learning for dental researchers. ‘Critical appraisal guidelines for assessing the quality and impact of user involvement in research’ (Wright et al., 2010) is also a useful resource to refer to when planning to use PPI.

However, PPI can be as simple as asking patients to contribute as authors to papers by providing a layperson’s perspective as demonstrated in this paper, and PaPE could be a social media post of a research paper accompanied by a plain language summary. Currently, to our knowledge, no orthodontic journals published in English provide plain language summaries of research articles for sharing via their website or other platforms, such as social media. A plain language summary example has been provided for this paper (Box 4). During the preparation of this paper the Editor of the *Journal of Orthodontics* was contacted to discuss whether plain language summaries may be considered in the future. The Editor expressed interest in this, and it is now being explored further.

**Box 4.** Plain language summary for this paper.

**Background** Patient and public involvement (PPI) is about patients and the public being partners in research rather than ‘subjects’. For example, patients can help decide which topics should be researched in order to make research more relevant to them. Patient and public engagement (PaPE) is a term used to describe activities which encourage the sharing findings and benefits of research with the patients/the public. For instance, posting research findings on social media accompanied by a summary designed for easy reading by patients and the public. Patient and public involvement and engagement (PPIE) is the collective term for PPI and PaPE. PPIE is important because healthcare research is designed to benefit patients and the public. Therefore, they should have a say and be involved in research studies. They should also be made aware of the type of research that is being carried out for their benefit. The use of PPIE makes for higher-quality and more ethical research. Research can be funded by different organisations called funding bodies. Researchers often apply for money (grants) from these organisations because certain types of research are not feasible without financial backing. Researchers who want to publish their research in scientific journals must follow the guidelines for authors written by each journal. **What did we do?** This study looks at PPIE in orthodontic research in three areas: 1. Whether authors are reporting their use of PPIE in their published research articles. We looked at all research papers published between September 2018 to September 2019 in four major orthodontic research journals. The authors of each paper were also emailed to find out if they had indeed used PPIE but may have omitted the report from their published article. 2. We looked at nine UK healthcare-based funding bodies to see if they include requirements for PPI and PaPE in order to be eligible for receiving a grant. 3. We looked at whether journals’ guidelines for authors include use of PPIE as something which should be included to make an article more likely to be published. **What did we find out?** There is a great lack of reporting of PPIE in orthodontic research articles. Replies from authors, although limited in number, also suggest lack of use of PPIE in research, which could explain why there is a lack of reporting. Funding bodies did not necessarily require PPIE for funding to be awarded. Journals’ guidelines for authors did not ask for any inclusion of PPIE use. **What do we suggest?** Researchers may be encouraged to increase their use and reporting of PPIE in the following ways: • Creating PPIE groups. • More funding bodies requesting use of PPIE before awarding money to research projects. • Including encouragement of PPIE use in journals’ guidance for authors.

Requirements for using and reporting PPIE by research bodies was found to be variable and while a number of funders request evidence of PPI in applications for funding, it is unclear how PPIE activity throughout the research is monitored. An example of guidance for PPI in research funding applications from HCRW is provided below.

‘Applicants should state in 2000 words:

- How they have involved and engaged public partners in the development of the application and the benefits this is expected to yield.
- Provide descriptions of the experience or area of activity of the public.
- Outline the activities in which they have been involved.
- The reasons for taking the approach.
- Explain how this involvement has/has not influenced or changed the research application.
- If the public were not involved in identifying the research topic and preparing the application, or if there are no plans to for active involvement, then there must be an explanation given for why this is not thought necessary.’ (Research for Patient and Public Benefit Wales [RfPPB], 2019).

This is useful for researchers for PPI during planning research but does not necessarily promote ongoing PPI throughout the research delivery and dissemination.

One challenge, highlighted by NH, is how to effectively recruit, train, support and retain PPI contributors. A review by Domecq et al. (2014) found most studies reported the positive effects of PPI but some patients involved in research became frustrated at the time required for training, attendance and transportation. However, with the advent of online video-conferencing platforms such as Zoom and Microsoft Teams, the cost of training individuals who reside in different parts of the country and travel may become less of an issue. It may be argued that patients and the public who contribute to PPI may not be representative of the population the research is being designed to benefit. They may have particular motivations for being involved with a research team and thus have an influence on steering the research in a particular direction. Researchers should take this into account when employing the use PPI. Similarly, researchers should think of ways to engage patients from a variety of different backgrounds rather than simply those who may be easiest to target when planning PaPE.

It is acknowledged that this study was limited to four orthodontic-specific journals, so orthodontic research published elsewhere was not considered and may provide different results. The lack of replies from authors regarding whether they actually employed the use of PPIE and simply did not report it may have been due to a number of factors. These include outdated contact details, language barriers, a lack of interest in PPIE and lack of use of PPIE, meaning they are less motivated to respond or the fact that this is a busy and difficult time period with regards to the COVID-19 pandemic.

## Conclusions

This study found that there is currently:

1. A lack of reporting of PPIE in orthodontic research.
2. Variability in the requirements of funding bodies for researchers to include PPIE in funding applications and throughout the research process.
3. No stipulation in journals’ instructions for authors for reporting PPIE or provision of a plain language summary.
